# Blood Pressure Regulation Evolved from Basic Homeostatic Components

**DOI:** 10.3390/biomedicines9050469

**Published:** 2021-04-25

**Authors:** Alon Botzer, Yoram Finkelstein, Ron Unger

**Affiliations:** 1 The Mina & Everard Goodman Faculty of Life Sciences, Bar-Ilan University, Ramat Gan 5290002, Israel; alon.botzer@gmail.com; 2 Neurology and Toxicology Service and Unit, Shaare Zedek Medical Center, Jerusalem 9103102, Israel; yoram.finkelstein@gmail.com

**Keywords:** blood pressure, homeostasis, evolution, gene orthologs

## Abstract

Blood pressure (BP) is determined by several physiological factors that are regulated by a range of complex neural, endocrine, and paracrine mechanisms. This study examined a collection of 198 human genes related to BP regulation, in the biological processes and functional prisms, as well as gene expression in organs and tissues. This was made in conjunction with an orthology analysis performed in 19 target organisms along the phylogenetic tree. We have demonstrated that transport and signaling, as well as homeostasis in general, are the most prevalent biological processes associated with BP gene orthologs across the examined species. We showed that these genes and their orthologs are expressed primarily in the kidney and adrenals of complex organisms (e.g., high order vertebrates) and in the nervous system of low complexity organisms (e.g., flies, nematodes). Furthermore, we have determined that basic functions such as ion transport are ancient and appear in all organisms, while more complex regulatory functions, such as control of extracellular volume emerged in high order organisms. Thus, we conclude that the complex system of BP regulation evolved from simpler components that were utilized to maintain specific homeostatic functions that play key roles in existence and survival of organisms.

## 1. Introduction

The multifactorial nature of BP regulation involves many genes with a widespread distribution across numerous cellular subsystems, posing significant challenges in the effort to decipher its complex mechanisms [1]. Here we implement a comparative approach to review the phylogenetic history of the BP system via an analysis of BP associated genes and their orthologs in 19 organisms, enabling us to gain an evolutionary perspective of the development of the system.

Current evidence proposes that the blood vascular system initially emerged in an ancestor of the tripoblasts over 600 million years ago, as a means to withstand the time-distance constraints of diffusion [2]. This has advanced over the course of evolution and expanded to a more complex “machinery” to support the functional requirements of high order organisms.

We suggest that the most fundamental organismal function of this machinery is to preserve the internal milieu, namely, to maintain a stable internal environment concerning temperature, electrolytes and water concentrations, as external conditions perpetually change. Internal environment, or “milieu intérieur” is a concept formulated by Claude Bernard, postulating that “the stability of the internal environment (the milieu intérieur) is the condition for the free and independent life”. This is the fundamental principle of homeostasis, a term coined later by Walter Bradford Cannon.

Claude Bernard also stated: “The constancy of the environment presupposes a perfection of the organism such that external variations are at every instant compensated and brought into balance. In consequence, far from being indifferent to the external world, the higher animal is on the contrary in a close and wise relation with it, so that its equilibrium results from a continuous and delicate compensation established as if the most sensitive of balances” [3].

This work has utilized bioinformatic tools and data mining techniques to investigate the BP system and elucidate the major homeostatic roles it played in the physiology of the corresponding organisms. In fact, we went back, tracing the evolution of genes that are involved in human BP regulation.

## 2. Materials and Methods

### 2.1. BP Genes, Orthologous Proteins and Target Organism Selection

**Figure 1:**
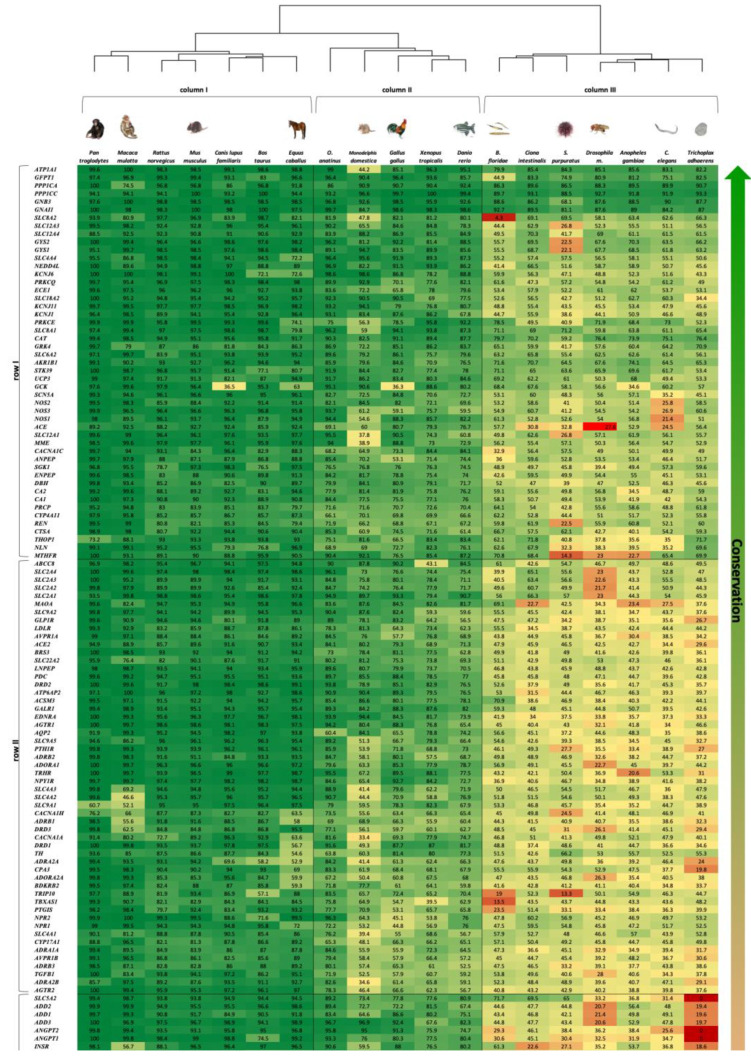

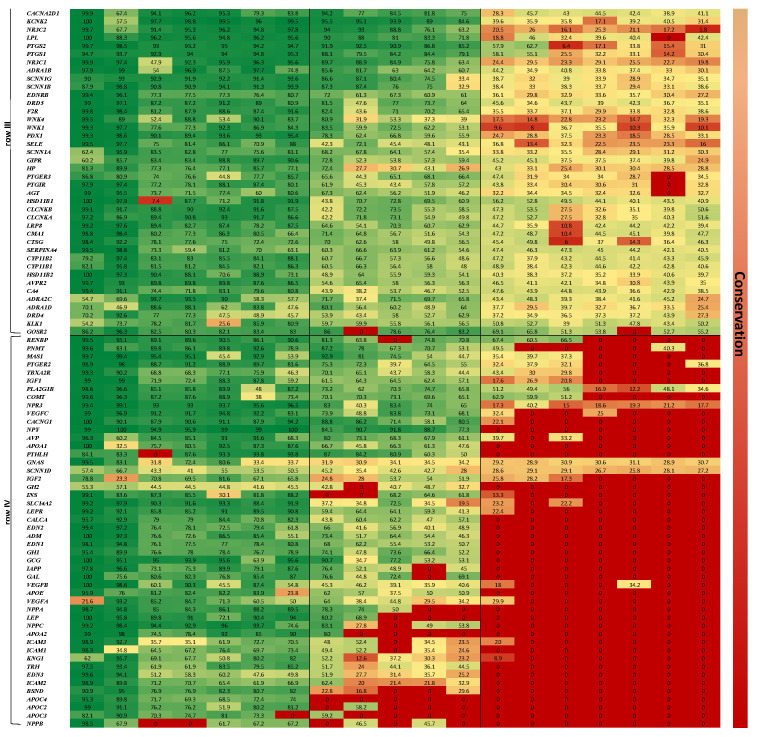
Heatmap displays the hierarchical clustering of similarity percentage scores between human BP genes (vertical axis) and their orthologs in 19 organisms (horizontal axis). Green color denotes the more conserved genes across the 19 organisms, aligned at the top of the map (see conservation scale arrow to the right of map). A phylogenetic tree describing the developmental order is placed above the map.

This study investigated a set of 198 genes associated with the BP regulation system. These genes were obtained and identified by means of bioinformatic analyses performed previously by Botzer et al. [4]. A set of orthologous proteins pertaining to these genes, from 19 organisms (see Figure 1) were obtained from two different sources—STRING and InParanoid.

STRING—Search Tool for the Retrieval of Interacting Genes/Proteins, (http://string-db.org/ (accessed on May 2017)) is a meta-resource that aggregates most of the available information on protein–protein associations and includes both direct physical interactions and protein interactions derived from literature. Since version 9.1, it contains pre-computed orthology relations imported from the eggNOG database [5]. eggnog—evolutionary genealogy of genes: Non-supervised Orthologous Groups, (http://eggnogdb.embl.de (accessed on May 2017)) is a public resource that provides Orthologous Groups (OG’s) of proteins at different taxonomic levels. It provides pairwise orthology relationships within OG’s based on analysis of phylogenetic trees. Protein sequences from the selected organisms were extracted and used to compute an all-against-all pair-wise similarity matrix. The comparison uses Smith-Waterman alignments and computational adjustments of the scores, as in BLAST, to prevent spurious hits between low-complexity sequence regions [6]. InParanoid (http://InParanoid.sbc.su.se (accessed on February 2018))—gathers proteomes of completely sequenced eukaryotic species and calculates pairwise ortholog relationships among them. This tool implements a two-pass BLAST approach that makes use of high-precision compositional score matrix adjustment, but avoids the alignment truncation that sometimes follows [7]. Both tools demonstrated similar results, but we decided to pursue analyses with data retrieved from STRING, which included a more comprehensive and updated coverage.

We then composed an organism-to-protein similarity map, consisting of similarity scores between the 198 human BP protein sequences and their ortholog proteins in the 19 target organisms, as obtained from STRING. This map was produced by calculation of pairwise sequence alignment, using EMBOSS Needle Global Alignment tool (https://www.ebi.ac.uk/Tools/psa/emboss_needle/ (accessed on February 2018)). This tool creates an optimal global alignment of two sequences using the Needleman-Wunsch algorithm, returning a value representing the percent of similarity between sequences, enabling homology to be inferred and the evolutionary relationship between the sequences studied [8].

Target organism selection was made with the intention to create a span of a wide variety of complexities—starting with the multicellular organism *T. adhaerens* as the simplest, going through flies, nematodes, fish, amphibians and eventually complex organisms as primates and other mammals (see Figure 1 for a complete list of organisms). We gave emphasis to well-researched model organisms, displaying sufficient and reliable orthology data, as well as extended annotations derived from established biological and genetic knowledge.

### 2.2. Creation of a Similarity Map and Hierarchical Clustering

The organism-to-protein similarity map enabled us to implement a two-way hierarchical clustering on the similarity matrix scores, also known as hierarchical cluster analysis.

Hierarchical clustering is an algorithm that recursively merges objects based on their pair-wise distance. Neighboring objects are merged first, while objects farthest apart are merged last. The ultimate result is a set of clusters, where each cluster is distinct from each other cluster, and the objects within each cluster are considerably similar to each other. The main output of hierarchical clustering is a dendrogram, which shows the hierarchical relationship between the clusters. This calculation provided us with clusters of organisms on one axis, and of BP proteins in the other axis, suggesting many interesting conclusions relating to the evolutionary order by which the circulatory system has evolved. Hierarchical clustering was performed using the Broad Institute Morpheus tool (https://software.broadinstitute.org/morpheus/ (accessed on May 2018)), implementing Euclidian distance metric and the Complete linkage method on both axes, emphasizing the maximum of the between-cluster dissimilarities [9].

### 2.3. Functional and Tissue Expression Enrichment Analyses

For a detailed in-depth analysis, we chose five organisms that essentially reflect the entire span of complexities of the 19 organisms that appear in the heatmap—*Homo sapiens*, *Mus musculus*, *Danio rerio*, *Drosophila melanogaster*, and *C. elegans*.

For each organism, we investigated the resulting clusters of orthologous proteins that were obtained from the hierarchical cluster analysis. These analyses consisted of functional GO term annotations (biological processes) as well as organ/tissue expression enrichment patterns. This was carried out by a set of tools, some of which specialize in human and mouse data, while others focus on other organisms. Table 1 describes the list of the tools we utilized.

**Figure 2:**
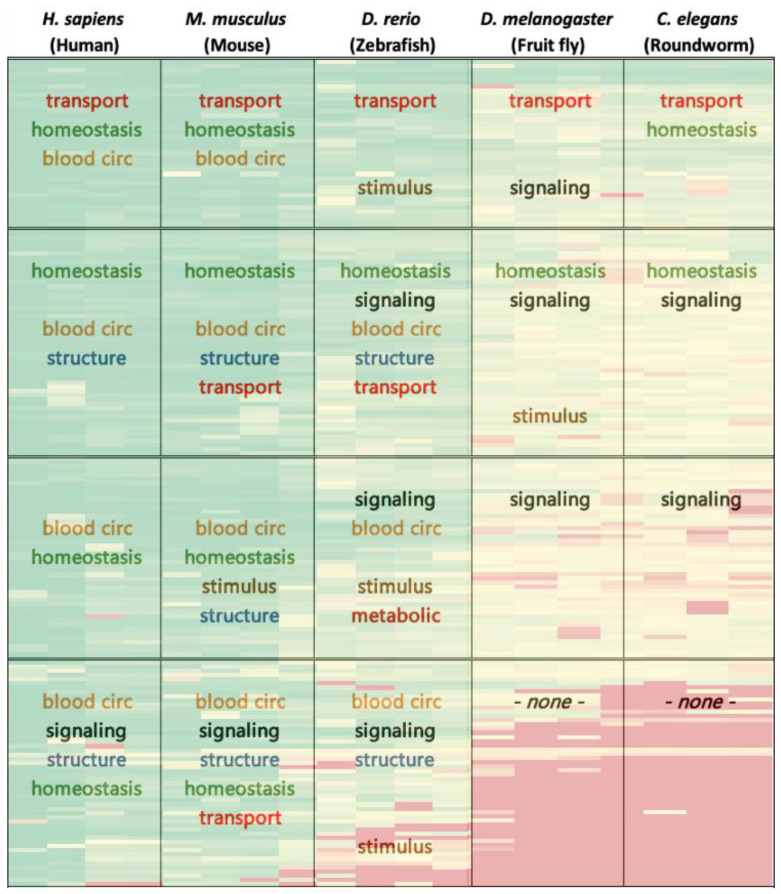
Demonstrates a summary of functional analysis results overlaid on heatmap of Figure 1. In each box, results pertain to the enriched biological processes for the respective genes-organisms of the cluster. For ease of interpretation, the colored text represents biological process families, reflecting their distribution throughout the map. For full functional analysis results please refer to Supplementary Table S1.

GO term enrichment tools (primarily GeneOntology) are used to evaluate characteristics of sets of genes by comparing the frequency of GO terms in the sample gene set with the frequency of the same set of GO terms in a reference set, usually a whole genome. The tools apply the binomial test to identify over or under-represented terms in the sample gene set compared to the reference genome set. The default parameters also apply a Bonferroni correction for multiple comparisons. A similar principle is implemented in the tissue enrichment analyses tools utilized for the different organisms, as specified in Figure 2. Since GO is roughly hierarchical, with “child” terms being more specialized than their “parent” terms, we were compelled to elect the terms in the 4–5 orders of the hierarchy in each cluster-species, in order to obtain broad definitions that enable comparison between the vast span of organisms in the analysis. As a control, the same GO term functional analysis was performed 30 times on randomly selected sets of 200 genes. This random analysis has not yielded any significant enrichment results, emphasizing the validity of the results for the set of blood pressure genes.

## 3. Results

The heatmap representing the results of the two-way clustered similarity map is presented in Figure 1. This map has yielded three clusters in the organism axis (columns I to III) and four clusters in the gene axis (rows I to IV).

The order by which the organism axis has aligned as a result of the clustering process is consistent with the natural known developmental order: (1) column I—high complexity mammals—from *Pan troglodytes* to *Equus caballus*, (2) column II—medium complexity—from *O. anatinus* to *Danio rerio*, (3) column III—low complexity—*B. floridae* to *T. adhaerens*. This order, presented in Figure 1 as a phylogenetic tree, is consistent with the developmental order of the blood circulatory mechanism in respect to the affiliated genes; 4—chamber heart system in high-order mammals and avians, 3 and 2—chamber heart in fish and amphibians, diffusion and hemolymph-based systems in insects and simple multi-cellular organisms.

The gene axis was aligned in four clusters, highlighting the more conserved genes across the 19 organisms at the top of the map (row I). The least conserved genes are shown at the bottom of the map (row IV), depicting a majority of zero value similarity scores, due to absence of orthologues genes in low complexity organisms. It is also amenable to discern that row I harbors genes associated with more basal functions, as ion transport, which is assumed to be ancient, hence appears in all organisms; while genes that are linked to a more complex regulatory functions, as control of extracellular volume or endothelial vasoconstriction appear in row IV, hence emerge in more recent organisms only.

Functional gene ontology (GO) term annotation (a statement regarding the function of genes) analysis demonstrates that homeostasis is the most prevalent biological process across most heatmap clusters. Transport processes are significant in row I, while blood circulation and vasculature structure are prevalent in columns I and II. Signaling processes appear most in row IV of columns I and II, rows I—III of columns II and III. Response to stimulus is mainly displayed in column II and slightly in columns I and III. Figure 2 demonstrates a summary of the functional analysis results for the various organisms investigated, displaying the enriched biological processes for each cluster. For full functional analysis results please refer to Supplementary Table S1.

**Figure 3:**
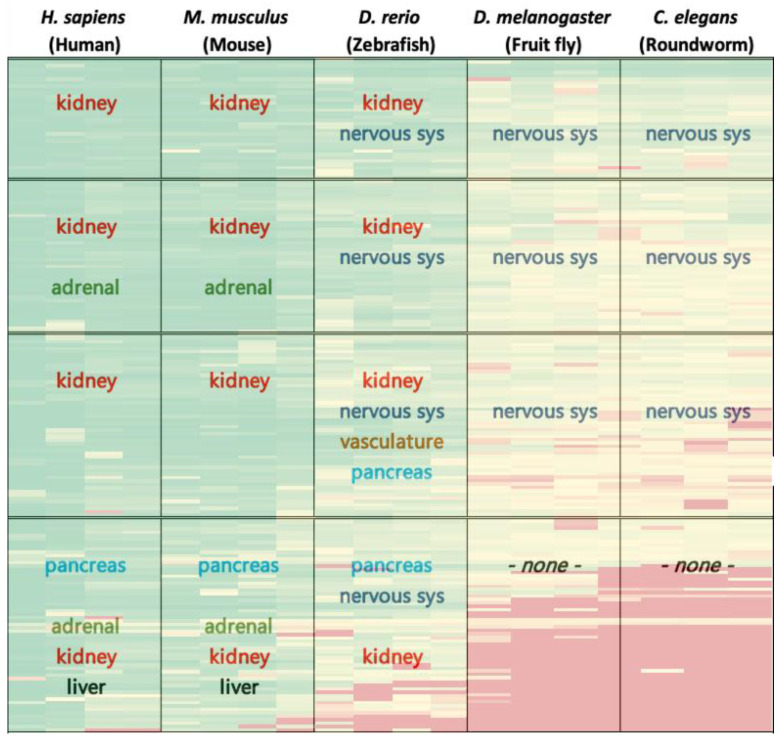
Demonstrates a summary of expression analysis results overlaid on heatmap of Figure 1. In each box, results pertain to the enriched organ/tissue expression for the respective genes-organisms of the cluster. For ease of interpretation, colored text represents tissue/organ types, reflecting their distribution throughout the map. For full functional analysis results please refer to Supplementary Table S2.

Tissue and organ expression enrichment analyses reveal that by and large genes in column I clusters are abundant in the kidney and somewhat in the adrenal gland, pancreas, and liver. In column II, gene expression is evident mainly in the kidney and nervous system, and slightly in the pancreas. This expression pattern changes significantly for column III, where gene expression is clearly noticeable exclusively in nervous system tissues. Figure 3 demonstrates a summary of tissue expression for the various organisms investigated. For full expression analysis results please refer to Supplementary Table S2. Altogether, the functional and expression results of the analysis in zebrafish display the summation of results of analyses in the higher and lower order species. This reflects the unique spot of zebrafish in ontogeny that well supports the rationale to exploit zebrafish as a model organism in biological research [10].

## 4. Discussion

Regulation of BP is a complex systemic mechanism due to numerous physiological elements, involving: pressure-volume regulation, which is tightly related to pressure-natriuresis [11], rapid control of vessel resistance by the central nervous system (CNS), specifically modulated by both sympathetic and parasympathetic nervous system—the two branches of the autonomic nervous system (ANS) [12], neurotransmitters (e.g., noradrenaline (NA), adrenaline) and hormones (e.g., angiotensin) as well as the long-term activity of the renin-angiotensin-aldosterone-system (RAAS). These mechanisms are efficient at maintaining BP within a normal physiological range at Systolic BP of <120 mmHg and Diastolic BP of <80 mmHg [13].

This study examined a collection of 198 human genes related to BP regulation in the biological processes and functional prisms, as well as gene expression in organ and tissue perspective. In this context, it is unavoidable that the question of homeostasis will arise. This observation, in conjunction with the orthology analysis we performed in 19 target organisms along the phylogenetic tree, evokes numerous interesting conclusions and further questions.

Homeostasis sustains the “internal milieu” of an organism’s cells, tissues, organs, and whole body, within limits that are compatible with survival. This internal milieu reflects the composition of the primordial ocean, first and foremost its electrolyte and water balance [14,15]. Homeostasis in homeothermic land-dwelling organisms is profoundly developed since their fluid balance, blood pH, oxygen tension and particularly their body temperature must be maintained within tight boundaries amid all conditions of their life cycle and in all their habitats. Homeostasis will tend to stabilize BP, maintaining it at a steady state.

The term stress defines any stimulus or succession of stimuli of such magnitude as to tend to disrupt the homeostasis of the organism. When mechanisms of adjustment fail or become incoordinate or disproportionate, the stress should be referred to as an insult. The response of the organism to stress depends largely on biochemical and physiological homeostatic mechanisms [16]. One of the most important mechanisms for maintaining homeostasis is the negative feedback system; if a physiological disturbance occurs, the body will counteract the disturbance via a negative feedback mechanism and attempt to return the body to its normal steady state.

The more complex the organism, the more complex its adaptation to the ever-changing environmental conditions. Biological systems may undergo functional plasticity in the effort to adapt. This is the mainstay of evolutionary progression [17].

The results of this study may elucidate the diversity of the modulating response mechanisms during phylogenesis: from the response of primitive organisms to physical stimuli (such as osmotic or heat stressors) to the complex endocrine and neurobehavioral stress response to both physical, mental, and cognitive challenges in human.

### 4.1. The Function of the CNS in the Maintenance of Homeostasis

The CNS is the organ that orchestrates the response to stressful stimuli through the autonomic, neuroendocrine, and immune systems, as well as through neurobehavioral responses such as fight-or-flight response. Adaptation to stress and to changing environmental conditions involves neural and humoral mediators: neurotransmitters and neuromodulators, hormones and cytokines of the immune system. The goal of this adaptation is to maintain homeostasis and promote survival of the organism [18].

The stress responses include primarily the activation of the mesolimbic system, the hypothalamo-pituitary-adrenal (HPA) axis and the ANS. The dopaminergic (DA-ergic) activity in the septo-hippocampus is a mainstay of the mesolimbic stress response, due to its close relationship with the hypothalamus (autonomic control), midbrain reticular formation (arousal) and multiple sensory pathways (attention). A DA-ergic pathway originates in the ventro-medial tegmentum of the midbrain and projects to the lateral septal nuclei with abundant indirect interconnections with the cholinergic nucleus of the medial septum, forming a final common pathway of the neural, psychic and endocrine stress response. Indeed, time honored studies corroborate the major importance of catecholamines in the ANS and of the glucocorticoids (GCs) in the adrenal cortex, as well as the CNS septo-hippocampal DA-ergic and cholinergic systems in the stress response. These complex mechanisms of adaptation are critical in the control of homeostasis [17,19]. Additionally, the mesolimbic pathway transports dopamine (DA) from the ventro-medial tegmentum to the nucleus accumbens and amygdala. The nucleus accumbens is found in the ventral medial portion of the striatum and is believed to play a role in reward, desire, and the placebo effect. The amygdala is a key component of the limbic system and is associated with emotion. Unabated DA-ergic stimulation (the absence of negative feedback) has been postulated to be associated with disorders such as binge eating or drug addiction [20,21].

The neuroendocrine system integrates the functions of two major control systems: the nervous system and the endocrine system. Thus, both internal and external fluctuations are monitored by the nervous sense organs, the neural signals of which are processed by the CNS and converted to endocrine outputs.

Furthermore, associated with the neuroendocrine system are distinct circumventricular fenestrations in the blood-brain barrier, mainly the chemoreceptor trigger zone (CTZ) in the area postrema of the medulla oblongata. The CTZ allows hormones and neurotransmitters to enter directly from the blood to the cerebrospinal fluid (CSF), hence enabling the feedback mechanisms with the neurosecretory system itself. 

The hypothalamus plays a principal role in the neuroendocrine control system, which together with its connections to the pituitary gland comprise the hypothalamic pituitary system—a neurosecretory system that produces releasing hormones (or releasing factors), emitting them to the pituitary gland. Important pituitary hormones are the adrenocorticotropic hormone (ACTH), which controls the adrenal cortex as well as growth hormone-inhibiting hormone (GHIH), which regulates the endocrine system and affects neurotransmission. Moreover, upper neurons of the ANS are located in the hypothalamus and control the adrenal medulla, an additional crucial endocrine gland. These roles of the CNS in the maintenance of homeostasis are dynamic, empowering the organism to function efficiently.

As seen in the Results section and in Figure 3, the adrenal gland and pancreas are strongly pronounced in human and mouse in rows II–IV, while the CNS and brain manifest rows I–III in fruitfly and roundworm. Interestingly, in the zebrafish gene enrichment in the CNS and the brain are prominent in all clusters, whereas the pancreas appears in rows III–IV, emphasizing the clear homology of these anatomic properties among the various species. This may suggest that organs that are enriched in expression of highly conserved genes across species reflect shared ancestry.

### 4.2. The Role of the Adrenal Glands

The adrenal gland comprises two endocrine tissues that differ in function and embryonal origin: The adrenal cortex, a steroidogenic tissue that evolved from the coelomic epithelium; and the adrenal medulla, a catecholamine producing tissue composed of chromaffin cells. The medulla derived from the neuroectoderm, which migrated to the adrenal gland blastema.

The secretion of the adrenal cortex hormones is controlled by ACTH in a closed loop feedback system. It produces numerous steroids secreted in widely varying amounts; these are classified as mineralocorticoids (MCs) and GCs including sex steroids. The main MC is aldosterone, which is the most potent steroid affecting active transport of sodium ions across membranes and thus is crucially important in maintaining electrolyte balance and as a result, in BP homeostasis. Cortisol is the principal GC in human and most mammalian primates and is quantitatively the main secretory product of the adrenal cortex.

The medulla is basically a modified sympathetic ganglion that converts tyrosine into DA, NA, and adrenaline, which are secreted in postsynaptic response to direct neural inputs. These inputs originate in the CNS (cortical cerebral areas) and are transmitted to the adrenal medulla via cholinergic preganglionic sympathetic neurons [22]. 

As noted, the primordium of the adrenal cortex coincides with the mesonephric blastema. It develops within the kidney in fish and on the kidney in amphibians. The kidneys of both fish and amphibians are of mesonephric origin [23].

In fish, chromaffin and cortical adrenal cells are not placed in proximity, but rather intermingle. The interrenal cells, that correspond to the adrenal cortex in mammals, from which GCs and MCs are secreted, do not encapsulate the chromaffin cells, which are the functional equivalent of the adrenal medulla. NA is the main catecholamine stored by the chromaffin cells, albeit variable quantities of adrenaline can also be present.

Amphibians show a closer relationship between cortical and chromaffin cells. The adrenals of amphibians lay on the ventral side of the kidneys, as the mesonephros—the analogue of the mammalian kidney, preserving the contact between the two organs during ontogenesis [24].

In mammals, where the evolution of the excretory system leads to the development of the metanephros, adrenal cortical cells completely surround the chromaffin cell mass—namely the medulla—and are grouped together to form the adrenal gland. Traces for this can be seen in our detailed expression analysis results (Supplementary Table S2), where row I–II are expressed in the adrenal glands of mammals, and in chromaffin cells and mesonephros of the zebrafish.

### 4.3. Nervous Regulation of the Cardiovascular System

The circulation is regulated partly by intrinsic and local mechanisms in each tissue and prominently by the nervous system, the action of which is extremely rapid and comprehensive. All the vascularized tissues in vertebrates are supplied with sympathetic nerve fibers. Sympathetic stimulation to the arterioles increases the resistance, thus changing the blood flow through the tissues. Sympathetic stimulation of the larger vessels decreases their blood volume. In a similar manner, cardiac output and BP are remarkably increased by increased sympathetic activity. Both heart rate and stroke volume (the two components of cardiac output) alongside vascular resistance are the main determinants of BP. 

The internal environment is monitored by sense organs and organelles: chemoreceptors sensitive to the partial oxygen pressure in the arterial blood, mechanoreceptors sensitive to blood pressure, and chemoreceptors within the central nervous system itself sensitive to hydrogen ion concentration or to various hormones. The input from the sense organs and organelles is transmitted to the CNS, in which it is processed and from which the appropriate outputs are sent to the effectors—muscles and glands. Above all, the vasomotor center (VMC)—in fact a network of neurons within the medulla oblongata, regulates BP and other homeostatic functions by a dual action: vasoconstriction and vasodilation.

Tonic adrenergic discharge to the arterioles sustains arterial pressure, while fluctuations in this tonic discharge constitute the mechanism affecting the feedback regulation of BP carried out by the carotid sinus (baroreceptor) and the carotid body (chemoreceptor that modulates the cardiovascular and respiratory systems via sympathetic tone). The effects of this discharge are of substantial importance in the preparation of the organism to endure emergency. 

### 4.4. Catecholamines and BP Homeostasis

Dopamine (DA), noradrenaline (NA), and adrenaline (known as catecholamines) are physiologically active molecules, acting both as hormones and neurotransmitters, crucial for the sustainment of homeostasis via the ANS. This work considered DA receptor genes (DRs), (DR1-like subtype—*DRD1*, *DRD5*, and DR2-like—*DRD2*, *DRD3*, *DRD4*) and adrenergic receptor genes (ARs), (adrenoceptors type alpha—*ADRA1A*, *ADRA1B*, *ADRA1D*, *ADRA2A*, *ADRA2B*, *ADRA2C*, and beta—*ADRB1*, *ADRB2*, *ADRB3*) appearing in rows II–III, all which play key physiological roles in the nervous and cardiovascular systems, specifically in BP regulation.

Almost all vasomotor nerves are adrenergic. The alpha-adrenoceptors are preeminent in innervation of vascular smooth muscles and also in the lower urinary tract. In the myocardium, beta1-adrenoceptors predominate and stimulate the rate and contractility. In both cases, neurotransmitter NA exerts its physiologic effects by binding to alpha ARs, whereas adrenaline reacts with both alpha and beta ARs, causing vasoconstriction and vasodilation, respectively. Generally, it is the alpha1-AR subtype, which is situated postsynaptically in smooth muscles, that causes vasoconstriction of blood vessels when stimulated. Sympathetic overactivity in hypertension results in an excess stimulation of postsynaptic alpha1 ARs [25].

DA, a major neurotransmitter in both CNS and PNS holds an essential function in the homeostatic control of BP, by regulation of vascular smooth muscle contraction, epithelial sodium transport, and reactive oxygen species (ROS) production. It is considered a major player in homeostatic regulation of extra-cellular fluid (ECF) volume and BP due to the vast effect it induces on renal hemodynamics as well as humoral agents such as catecholamines, aldosterone, renin, vasopressin and endothelin (e.g., DA inhibits NA release and acts as a vasodilator at normal concentrations by activation of D2 receptors; decreases aldosterone secretion via activation of D3 receptors, while significantly enhancing renin secretion by activation of D1 receptors).

DA also adjusts sodium and fluid intake by means of activities within the gastrointestinal tract and CNS, primarily by regulation of the cardiovascular control centers in the brain stem [26]. Each DA receptor subtype participates in BP regulation by specific mechanisms for the subtype; *DRD2* and *DRD5* act within the CNS and PNS.

The mesolimbic DA system is implicated in diurnal profiles of the mean BP. A clear dip in the mean BP and heart rate occurs during the resting period (e.g., nocturnal dip in human) [27]. An increase in arterial BP can be seen during the transition from non-REM to REM sleep, displaying phasic surges throughout REM sleep that derive from the physiological phase of paradoxical sleep. Furthermore, the mesolimbic DA system is involved in the increases in REM-associated BP fluctuations [28], a homeostatic adaptation to the fluctuating states of the different sleep and arousal phases. Since the mesolimbic DA system surges are involved in REM-associated increases in BP, the homeostatic effect is the result of a greater ability to deal with emotional stress. The pineal gland translates light signals received by the retina to the rest of the body, for example through the synthesis of the hormone melatonin, which is produced and released at night and helps to regulate the body’s metabolic activity during sleep. NA is involved in regulating this synthesis and release of melatonin in the pineal gland. DA through *ADRA1B-DRD4* and *ADRB1-DRD4* receptor heteromers inhibits the effects of NE, resulting in a decrease in the production and release of melatonin [29].

Although there are two classes of DA receptors, DR1-like and DR2-like, the natriuretic effect of DA is primarily mediated by DR1-like receptors [30]. During sodium loading, DR2-like receptors may contribute to the natriuresis [31]. Of the three DR2-like receptors it is likely that it is the *DRD3* receptor that interacts with the *DRD1* receptor because it is the major DR2-like receptor expressed in the renal proximal tubule and the thick ascending limb of Henle [32]. The *DRD1*, *DRD3*, *DRD4* receptors interact with the renin-angiotensin system (RAAS), affecting epithelial transport and control of secretion of multiple humoral agents and their receptors [33].

From the comparative perspective, our results show a high degree of conservation (>60%) for catecholamines in vertebrate species analyzed, especially in mammals, as well as in invertebrates such as *C. elegans*, *Drosophila melanogaster*, and *Ciona intestinalis* (~45%). 

The vertebrate DR1-like receptors are most closely related to the *DOP1* group members of *C. elegans* and *Drosophila melanogaster*, both responsible for upregulation of intracellular cAMP in the presence of DA. The vertebrate DR2-like receptors share the most homology with the *C. elegans CeDOP2* and the *Drosophila melanogaster DD2R*, sharing a similar response in the decrease of intracellular cAMP levels when treated with DA [34]. In *Drosophila melanogaster*, expression patterns of *DOP1* (an ortholog of the vertebrate D1-type DA receptors) were observed in mushroom body neurons, subesophageal ganglion, and in unpaired abdominal ganglia [35], while *DD2R* is expressed in the larval and adult nervous systems, and in cell groups that include peptidergic neurons [36]. In *C. elegans* it was shown that *CeDOP1* is expressed in mechanosensory neurons, motor neurons and the ventral nerve cord, as well as sensory support cells, which are glial-like cells that surround the sensilla (simple invertebrate sense organs that may take the form of a hair or bristle) [37], while *CeDOP2* is expressed in DA-nergic neurons, suggesting it may act as an autoreceptor [38].

### 4.5. The Kidney’s Role in Maintaining Homeostasis

Notably, genes in clusters of rows I–III are expressed in the kidney of zebrafish, mouse, and human. Their functional analysis demonstrated that homeostasis and transport are the primary biological processes that characterize these clusters, while signaling and stimulus are secondary.

The cells of the kidney contain many specialized ion channels and transporters, which act in concert to regulate volume and ionic concentration by absorption or secretion of ions into the urine [39]. The ion channels and transporters play essential roles in organelle to whole organism function. These roles range from regulation of cell volume, membrane excitability and pH, to control of systemic salt and water balance and behavior [40].

Our work specifically relates to several prominent gene families—solute carriers (primarily electroneutral potassium chloride cotransporters, glucose transporters, bicarbonate transporter proteins), calcium voltage-gated channels, sodium channels, epithelial chloride voltage-gated channels, potassium voltage-gated channels, DRs, and ARs, which are conserved across most species examined (similarity > 50%).

It is especially important to underscore the largest gene family we analyzed in this work (21 members)—Solute Carriers (SLCs), which are responsible for the regulation of various types of substances over the cell membrane. They typically rely on an ion gradient over the cell membrane as a mechanism for transportation [41]. Interestingly, all SLCs that are found in human are also found in *C. elegans* and *Drosophila melanogaster* and even some in *T. adhaerens* (*SLC8A2, SLC12A3, SLC12A4, SLC4A4, SLC6A2* >50% similarity), indicating that this superfamily is ancient and was present before the divergence of Bilateria (animals with bilateral symmetry).

Another noteworthy gene family we analyzed is the voltage-gated ion channel superfamily, comprising calcium, potassium, and sodium ion channels. As can be seen from the heatmap in Figure 1, *KCNJ1, KCNJ11, KCNJ6* are well conserved (>45%) in all 19 species, including *T. adhaerens*, along with *CACNA1C, CACNA1H, CACNA1A, CACNA2D1* (>40%) in all 19 species. Yu et al. suggested that this family has evolved from a bacterial ancestor channel and accumulated regulatory domains for ligand binding in the course of evolution. The resulting signaling mechanisms control most aspects of cell physiology, complex processes in the brain and movements of muscles, all of which allowed the development of complex multicellular organisms [42].

Multicellular organisms control their internal environment by altering either the electrolytes concentration (osmolality) or the extracellular volume, maintaining the acid-base balance. In mammals, the main site of this complex regulation is the kidney. 

The role of human kidney is maintaining the fragile equilibrium within the internal milieu, namely the ECF and the intra-cellular fluid (ICF). From the evolutionary point of view, the human kidney evolved from primitive mechanisms of the ionic and osmotic homeostasis in fish. The data presented in this work provide evidence that the genes that are implicated in these mechanisms appear in fish, but not in earlier stages of phylogeny.

The ion composition of the ECF is essentially identical in all animal species (including fish, amphibians, reptiles, and mammals) where the kidneys are utilized to regulate homeostasis [15]. Since salt concentration of the ocean increased over time, saltwater fish began to contain hypo-osmolar internal milieu and constantly exhausted water to the hyperosmolar environment. To endure this challenge, saltwater fish consume considerable amounts of water [43]; hence the acquired capability of fish to actively preserve the internal milieu is remarkable.

The zebrafish pronephros, which consists of two nephrons with glomeruli, contains two tubules that are analogous in many ways to the segments of the mammalian nephron [44]. The proximal straight segment of this nephric tubule displays a brush border-like columnar epithelial cells [45]. This segment plays a crucial role in reabsorption of electrolytes and small molecules through a glomerular filtration barrier [46]. This is the physiological principle that underlies the mammalian renal function.

Kidney functions are achieved both independently and in conjunction with other organs, particularly the endocrine system via various endocrine hormones, including, among others: renin (*REN*), angiotensin II, aldosterone, antidiuretic hormone (*ADH*), and two atrial natriuretic peptides (*NPPA*, *NPPB*) which are evolutionarily conserved. These natriuretic peptide hormones are secreted by atrial myocytes in response to stretch, angiotensin II stimulation, endothelin, and sympathetic (beta adrenergic) stimulation [47].

Blocking ion channels is an important part of anti-hypertensive pharmacotherapy. This is exemplified by the commonly used drugs—nifedipine, amlodipine, diltiazem, and verapamil which block the voltage-gated calcium channels of vascular smooth muscle cells, thus lowering BP; and amiloride that blocks the epithelial sodium channels at the distal convoluted tubule and the collecting duct, thereby inhibiting sodium-potassium exchange, while lowering BP independent of aldosterone [48,49].

### 4.6. Sensing the Environment in Order to Maintain Homeostatic Balance

An extensive network of sensory organs and organelles play an important role in maintaining homeostatic balance and monitoring the internal environment. These include: mechanoreceptors sensitive to BP, chemoreceptors that react to the partial oxygen pressure in the arterial blood and chemoreceptors within the CNS, which are sensitive to pH and to numerous hormones. Ion channels in the hypothalamus gauge the ECF osmolality and sodium concentration. Baroreceptors situated in numerous circulatory beds along with ion channels and various receptors within the distal tubule in the kidney nephron, regulate blood volume. The input from the sense organs and organelles is transmitted for processing to the CNS, from which appropriate outputs are forwarded to the effector tissues—muscles and glands. The volume effectors are the renin–angiotensin–aldosterone system (RAAS), alongside sympathetic nerve activation (SNA) as well as glomerular filtration rate and physical gradients along the nephron. Homeostatic control mechanisms maintain the balance between fluid gain and fluid loss. Body water homeostasis is mainly regulated through fluid ingestion. Sensory osmoreceptors, found primarily in the hypothalamus, detect changes in plasma osmolarity and contribute to fluid-balance regulation in the body. If the body is fluid deficient, increased plasma osmolarity is sensed by the osmoreceptors. When osmoreceptors detect high plasma osmolarity (a sign of low blood volume/dehydration), they send signals to the hypothalamus, which creates the sensation of thirst. This result also triggers an increase in ADH secretion, causing fluid retention by the kidneys and urine output to be reduced. Macula densa cells in the nephron’s ascending loop of Henle are another type of osmoreceptor. If the macula densa is stimulated by high osmolarity, the juxtaglomerular apparatus (JGA) releases renin into the bloodstream. The subsequent production of angiotensin II acts on the hypothalamus to cause thirst. Angiotensin II causes vasoconstriction and aldosterone release. Aldosterone increases expression of the nephron’s ATPase pumps resulting in increased water reabsorption through sodium cotransport [50].

### 4.7. RAAS Evolution

The RAAS is the primary volume-regulating pathway in mammals, acting as the central controller of blood pressure homeostasis in humans. The renin-angiotensin-aldosterone system emerged approximately 450 million years ago, in the Paleozoic era, as marine organisms moved to the land and endured a strong selection pressure to conserve salt and maintain volume homeostasis, which were crucial for survival [51]. In this work we have examined the sequence homologs for 20 human genes associated with RAAS pathway in 19 model organisms. Interestingly, homologs for all genes were present in all species from *Danio rerio* (Zebrafish) up to *Pan troglodytes* (Chimpanzee), with lowest similarity score of 46% for angiotensinogen (*AGT*). Since our data do not provide evidence for the presence of *AGT* homologs prior to *Danio rerio*, and due to its central role in the RAAS, this could indicate that the pathway emerged approximately with the appearance of zebrafish. This observation, in conjunction with the fact that zebrafish is the taxon with the most primitive juxtaglomerular apparatus [52], agrees with the physiological evidence regarding its emergence in bony fishes [53]. Our work reveals several orthologs for *ACE* and *ACE2* from *B. floridae* to *T. adhaerens*, representing primitive chordates, as well as insects and multicellular organisms, which lack most RAAS components and are characterized by an open circulatory system. No ortholog sequence was found in the *C. elegans*, a fact that challenges the advent and initial roles of the enzyme. Recent genomic data revealed the presence of orthologs in yet additional remote phyla, as placozoa and proteobacteria, suggesting that it emerged early in evolution. Thus, by and large, *ACE* exists from bacteria up to mammals, exhibiting conserved features such as structure, as well as molecular and biochemical characteristics. In higher order organisms, *ACE* exerts its effect mainly in the capillaries of the lungs, whereas the evolution of the respiratory system was a key step in the transition of the animal world from marine life to land habitation. Characteristics of mammalian *ACE* could therefore be the outcome of an extended course of evolutionary specialization of an ancient protease, the functions of which are not yet known [54].

Adrenal steroids, both MCs and GCs, influence almost all aspects of vertebrate development, as well as regulation of an extensive selection of physiological processes, including development, differentiation, reproduction, and homeostasis [24]. Their receptors belong to the nuclear receptor super-family of proteins, which are ligand-activated transcription factors, implicated in steroid activity in a myriad of physiological, morphological, and behavioral processes [55].

GRs and MRs encoded in human by the *NR3C1* and *NR3C2* genes respectively, are representatives of the two principal functional families of corticosteroid receptors in vertebrates. These have not been found in invertebrates, as confirmed in our work, where orthologs for *NR3C1* and *NR3C2* appear first only in *Danio rerio* with similarity >63%. The MR and the GR are known to have descended from the early corticosteroid receptor [56], which first appeared in jawless fish, at the base of the vertebrate lineage, while distinct MR first appeared in cartilaginous fish [57]. Interestingly, the ancient GR evolved through duplication, to develop into a GR due to two gene mutations [58]. It is hypothesized that this evolutionary process would have alleviated the difficulty to adapt to blood pressure elevation caused by increased gravitational effects of land habitation [59]. 

## 5. Conclusions

To conclude this study, it appears that the genes that are involved in BP regulation are primarily implicated in mechanisms associated with the maintenance of homeostasis. Evidence for this can be seen in the conserved genes across the entire range of organisms investigated, performing functions that are notably related to homeostasis, e.g., ion transport, response to stimulus and more, as well as expression patterns in tissues and organs that support homeostatic processes in the respective species. 

This work is merely an attempt to cover the broad scope of the evolutionary dissection of the genetic machinery behind BP regulation; nonetheless it highlights the importance of the comparative approach that presupposes that the comprehension of our evolutionary past can highly enlighten our understanding of the genetic background of common disease.

## Figures and Tables

**Table 1:** Tissue enrichment and functional annotation tools utilized in this work.

Tool	Link to URL	Content
GeneAnalytics	https://ga.genecards.org/ (accessed on February 2018)	Expression-based matching algorithm, provides gene annotations and enrichment in each specific tissue or organ in human or mouse.
GeneOntology	geneontology.org/ (accessed on February 2018)	Provides a computational representation regarding the functional enrichment of genes (proteins produced by genes) from a variety of organisms from human to bacteria.
g:Profiler	https://biit.cs.ut.ee/gprofiler/gost (accessed on October 2018)	Finds biological categories enriched in gene lists, functionality, conversions between gene identifiers and mappings to their orthologs.
TissueEnrich	https://tissueenrich.gdcb.iastate.edu/ (accessed on October 2018)	Defines tissue-specific genes using RNA-Seq data from the Human Protein Atlas, GTEx, and mouse ENCODE.
Bgee	https://bgee.org/ (accessed on November 2018)	A database used to retrieve and compare gene expression patterns in multiple animal species.
TSEA	http://genetics.wustl.edu/jdlab/tsea/ (accessed on October 2018)	Tissue specific expression analysis for human and mouse.
MouseMine	http://www.mousemine.org/mousemine/begin.do (accessed on November 2018)	Provides queries for anatomy and gene ontology enrichments as well as other analyses for mouse.
ZebrafishMine	http://www.zebrafishmine.org/begin.do (accessed on November 2018)	Searches for zebrafish related biological data including genes, proteins, tissues, and more.
ZEOGS	http://zeogs.scienceontheweb.net/index.html (accessed on November 2018)	Predicts sites of preferential expression of zebrafish gene sets.
FlyMine	http://www.flymine.org/ (accessed on November 2018)	Integrates many types of data for drosophila and other organisms. runs flexible queries for genes, proteins, ontology terms, and tissue enrichments.
FlyBase	http://flybase.org/convert/id (accessed on December 2018)	Curates and organizes a diverse array of genetic, molecular, genomic, and developmental information in drosophila.
WormBase	https://www.wormbase.org/tools/enrichment/tea/tea.cgi (accessed on December 2018)	Provides information concerning the genetics, genomics, and biology of *C. elegans* and related nematodes.
WormExp	http://wormexp.zoologie.uni-kiel.de/wormexp/ (accessed on December 2018)	Integrates all published expression data for *C. elegans*, runs flexible queries for gene enrichment analyses on selected gene sets.

## Data Availability

Data supporting reported results of this study can be found in publicly archived datasets as specified in links throughout the manuscript.

## Supplementary Materials

The following are available online at https://www.mdpi.com/article/10.3390/biomedicines9050469/s1, Table S1: GO term biological processes enrichment per cluster genes and organisms, Table S2: tissue/organ enrichment per cluster genes and organisms.

## Abbreviations

CNS	Central Nervous System
ANS	Autonomous Nervous System
RAAS	Renin Angiotensin Aldosterone System
DA	Dopamine
NA	Noradrenaline
CTZ	Chemoreceptor Trigger Zone
ACTH	Adrenocorticotropic Hormone
GC	Glucocorticoid
GR	Glucocorticoid Receptor
AR	Adrenergic Receptor
ECF	Extracellular Fluid
SLC	Solute Carrier
GO	Gene Ontology

## References

[1] Rossier B.C., Bochud M., Devuyst O. (2017). The hypertension pandemic: An evolutionary perspective. Physiology.

[2] Monahan-Earley R., Dvorak A.M., Aird W.C. (2013). Evolutionary origins of the blood vascular system and endothelium. J. Thromb. Haemost..

[3] Bernard C. (1974). Lectures on the Phenomena of Life Common to Animals and Plants.

[4] Botzer A., Grossman E., Moult J., Unger R. (2018). A system view and analysis of essential hypertension. J. Hypertens..

[5] Szklarczyk D., Morris J.H., Cook H., Kuhn M., Wyder S., Simonovic M., Santos A., Doncheva N.T., Roth A., Bork P. (2017). The STRING database in 2017: Quality-controlled protein-protein association networks, made broadly accessible. Nucleic Acids Res..

[6] Huerta-Cepas J., Szklarczyk D., Forslund K., Cook H., Heller D., Walter M.C., Rattei T., Mende D.R., Sunagawa S., Kuhn M. (2016). eggNOG 4.5: A hierarchical orthology framework with improved functional annotations for eukaryotic, prokaryotic and viral sequences. Nucleic Acids Res..

[7] Sonnhammer E.L.L., Ostlund G. (2015). InParanoid 8: Orthology analysis between 273 proteomes, mostly eukaryotic. Nucleic Acids Res..

[8] Madeira F., Park Y.M., Lee J., Buso N., Gur T., Madhusoodanan N., Basutkar P., Tivey A.R.N., Potter S.C., Finn R.D. (2019). The EMBL-EBI search and sequence analysis tools APIs in 2019. Nucleic Acids Res..

[9] Greenacre M.J., Primicerio R. (2013). Multivariate Analysis of Ecological Data.

[10] Lieschke G.J., Currie P.D. (2007). Animal models of human disease: Zebrafish swim into view. Nat. Rev. Genet..

[11] Guyton A.C., Hall J.E. (1996). Textbook of Medical Physiology.

[12] Walch L., Brink C., Norel X. (2001). The muscarinic receptor subtypes in human blood vessels. Therapie.

[13] Whelton P.K., Carey R.M., Aronow W.S., Casey D.E., Collins K.J., Dennison Himmelfarb C., DePalma S.M., Gidding S., Jamerson K.A., Jones D.W. (2018). 2017 ACC/AHA/AAPA/ABC/ACPM/AGS/APhA/ASH/ASPC/NMA/PCNA Guideline for the Prevention, Detection, Evaluation, and Management of High Blood Pressure in Adults: A Report of the American College of Cardiology/American Heart Association Task Force on Clinical Pr. Hypertension.

[14] Schulte K., Kunter U., Moeller M.J. (2015). The evolution of blood pressure and the rise of mankind. Nephrol. Dial. Transplant..

[15] Epstein F.H. (1999). The sea within us. J. Exp. Zool..

[16] Nesse R.M., Bhatnagar S., Young E.A. (2007). Evolutionary Origins and Functions of the Stress Response. Encyclopedia of Stress.

[17] Finkelstein Y., Koffler B., Rabey J.M., Gilad G.M. (1985). Dynamics of cholinergic synaptic mechanisms in rat hippocampus after stress. Brain Res..

[18] Dantzer R. (2018). Neuroimmune interactions: From the brain to the immune system and vice versa. Physiol. Rev..

[19] McEwen B.S., Nasca C., Gray J.D. (2016). Stress effects on neuronal structure: Hippocampus, amygdala, and prefrontal cortex. Neuropsychopharmacology.

[20] Blaess S., Stott S.R.W., Ang S.-L. (2020). The generation of midbrain dopaminergic neurons. Patterning and Cell Type Specification in the Developing CNS and PNS.

[21] Preedy V.R. (2016). Neuropathology of Drug Addictions and Substance Misuse.

[22] Lumb R., Schwarz Q. (2015). Sympathoadrenal neural crest cells: The known, unknown and forgotten?. Dev. Growth Differ..

[23] Grassi Milano E., Basari F., Chimenti C. (1997). Adrenocortical and adrenomedullary homologs in eight species of adult and developing teleosts: Morphology, histology, and immunohistochemistry. Gen. Comp. Endocrinol..

[24] Baker M.E. (2003). Evolution of adrenal and sex steroid action in vertebrates: A ligand-based mechanism for complexity. BioEssays.

[25] Michel M.C., Brodde O.E., Insel P.A. (1990). Peripheral adrenergic receptors in hypertension. Hypertension.

[26] Mannelli M., Pupilli C., Lanzillotti R., Ianni L., Serio M. (1990). Catecholamines and blood pressure regulation. Horm. Res..

[27] Sakata M., Sei H., Toida K., Fujihara H., Urushihara R., Morita Y. (2002). Mesolimbic dopaminergic system is involved in diurnal blood pressure regulation. Brain Res..

[28] Sei H., Morita Y. (1999). Why does arterial blood pressure rise actively during REM sleep?. J. Med. Investig..

[29] González S., Moreno-Delgado D., Moreno E., Pérez-Capote K., Franco R., Mallol J., Cortés A., Casadó V., Lluís C., Ortiz J. (2012). Circadian-related heteromerization of adrenergic and dopamine d4 receptors modulates melatonin synthesis and release in the pineal gland. PLoS Biol..

[30] Hussain T., Lokhandwala M.F. (1998). Renal dopamine receptor function in hypertension. Hypertension.

[31] Jose P.A., Asico L.D., Eisner G.M., Pocchiari F., Semeraro C., Felder R.A. (1998). Effects of costimulation of dopamine D1- and D2-like receptors on renal function. Am. J. Physiol..

[32] O’Connell D.P., Vaughan C.J., Aherne A.M., Botkin S.J., Wang Z.-Q., Felder R.A., Carey R.M. (1998). Expression of the dopamine D_3_ receptor protein in the rat kidney. Hypertension.

[33] Jose P.A., Eisner G.M., Felder R.A. (2003). Dopamine and the kidney: A role in hypertension?. Curr. Opin. Nephrol. Hypertens..

[34] Mustard J.A., Beggs K.T., Mercer A.R. (2005). Molecular biology of the invertebrate dopamine receptors. Arch. Insect Biochem. Physiol..

[35] Kim Y.C., Lee H.G., Seong C.S., Han K.A. (2003). Expression of a D1 dopamine receptor dDA1/DmDOP1 in the central nervous system of *Drosophila melanogaster*. Gene Expr. Patterns.

[36] Draper I., Kurshan P.T., McBride E., Jackson F.R., Kopin A.S. (2007). Locomotor activity is regulated by D2-like receptors in Drosophila: An anatomic and functional analysis. Dev. Neurobiol..

[37] Tsalik E.L., Niacaris T., Wenick A.S., Pau K., Avery L., Hobert O. (2003). LIM homeobox gene-dependent expression of biogenic amine receptors in restricted regions of the *C. elegans* nervous system. Dev. Biol..

[38] Suo S., Sasagawa N., Ishiura S. (2002). Identification of a dopamine receptor from *Caenorhabditis elegans*. Neurosci. Lett..

[39] Kuo I.Y., Ehrlich B.E. (2012). Ion channels in renal disease. Chem. Rev..

[40] Strange K. (2003). From genes to integrative physiology: Ion channel and transporter biology in *Caenorhabditis elegans*. Physiol. Rev..

[41] Hoglund P.J., Nordstrom K.J.V., Schioth H.B., Fredriksson R. (2011). The solute carrier families have a remarkably long evolutionary history with the majority of the human families present before divergence of bilaterian species. Mol. Biol. Evol..

[42] Yu F.H., Catterall W.A. (2004). The VGL-chanome: A protein superfamily specialized for electrical signaling and ionic homeostasis. Sci. STKE.

[43] Hirano T. (1974). Some factors regulating water intake by the eel, *Anguilla japonica*. J. Exp. Biol..

[44] Wingert R.A., Davidson A.J. (2008). The zebrafish pronephros: A model to study nephron segmentation. Kidney Int..

[45] Alpern R., Moe O., Caplan M. (2013). Seldin and Geibisch’s The Kidney.

[46] Carroll T.J., Wallingford J.B. (2003). Induction, Development, and Physiology of the Pronephric Tubules. Kidney.

[47] Man J., Barnett P., Christoffels V.M. (2018). Structure and function of the Nppa–Nppb cluster locus during heart development and disease. Cell. Mol. Life Sci..

[48] Baker E.H. (2000). Ion channels and the control of blood pressure. Br. J. Clin. Pharmacol..

[49] Botzer A., Finkelstein Y., Grossman E., Moult J., Unger R. (2018). Iatrogenic hypertension: A bioinformatic analysis. Pharmacogenom. J..

[50] Water Balance—Boundless Anatomy and Physiology. https://courses.lumenlearning.com/boundless-ap/chapter/water-balance/.

[51] Mansley M.K., Ivy J.R., Bailey M.A. (2016). ISN Forefronts Symposium 2015: The evolution of hypertension—Old genes, new concepts. Kidney Int. Rep..

[52] Sokabe H., Ogawa M. (1974). Comparative studies of the *Juxtaglomerular apparatus*. Int. Rev. Cytol..

[53] Nishimura H., Bailey J.R. (1982). Intrarenal renin-angiotensin system in primitive vertebrates. Kidney Int..

[54] Rivière G., Michaud A., Deloffre L., Vandenbulcke F., Levoye A., Breton C., Corvol P., Salzet M., Vieau D. (2004). Characterization of the first non-insect invertebrate functional angiotensin-converting enzyme (ACE): Leech TtACE resembles the N-domain of mammalian ACE. Biochem. J..

[55] Bentley P.J. (1998). Comparative Vertebrate Endocrinology.

[56] Thornton J.W. (2001). Evolution of vertebrate steroid receptors from an ancestral estrogen receptor by ligand exploitation and serial genome expansions. Proc. Natl. Acad. Sci. USA.

[57] Osório J., Rétaux S. (2008). The lamprey in evolutionary studies. Dev. Genes Evol..

[58] Bridgham J.T., Carroll S.M., Thornton J.W. (2006). Evolution of Hormone-Receptor Complexity by Molecular Exploitation. Science.

[59] Kvetnansky R., Lu X., Ziegler M.G. (2013). Stress-triggered changes in peripheral catecholaminergic systems. Adv. Pharmacol..

